# *In situ* determination of the extreme damage resistance behavior in stomatopod dactyl club

**DOI:** 10.1107/S1600577522001217

**Published:** 2022-03-14

**Authors:** Zheng Dong, Sen Chen, Himadri S. Gupta, Xiaoyi Zhao, Yiming Yang, Guangcai Chang, Jian Xue, Yiyang Zhang, Shengnian Luo, Yuhui Dong, Yi Zhang

**Affiliations:** a Beijing Synchrotron Radiation Facility, Institute of High Energy Physics, Chinese Academy of Sciences, Beijing 100049, People’s Republic of China; b University of Chinese Academy of Sciences, Beijing 100049, People’s Republic of China; c School of Materials Science and Engineering, Ministry of Education, Southwest Jiaotong University, Chengdu, Sichuan 610031, People’s Republic of China; d School of Engineering and Material Science, Queen Mary University of London, London E1 4NS, People’s Republic of China; e Chinese Spallation Neutron Source Science Centre, Dongguan, Guangdong 523808, People’s Republic of China; f State Key Laboratory of Nonlinear Mechanics (LNM), Institute of Mechanics, Chinese Academy of Sciences, Beijing 100190, People’s Republic of China

**Keywords:** stomatopod dactyl, *in situ* characterization, 3D crack evolution, fiber bridging, toughening mechanisms

## Abstract

The structure and mechanical properties of the stomatopod dactyl club have been studied extensively for its extreme impact tolerance, but a systematic *in situ* investigation on the multiscale mechanical responses under high-speed impact has not been reported. Here the full dynamic deformation and crack evolution process within projectile-impacted dactyl using combined fast 2D X-ray imaging and high-resolution *ex situ* tomography are revealed. The results show that hydration states can lead to significantly different toughening mechanisms inside dactyl under dynamic loading. A previously unreported 3D interlocking structural design in the impact surface and impact region is reported using nano X-ray tomography. Experimental results and dynamic finite-element modeling suggest this unique structure plays an important role in resisting catastrophic structural damage and hindering crack propagation. This work is a contribution to understanding the key toughening strategies of biological materials and provides valuable information for biomimetic manufacturing of impact-resistant materials in general.

## Abstract 2

Multiscale structural and mechanical characterization of stomatopod cuticle under *in situ* spherical projectile penetrating loadings. In this work the full dynamic crack evolutionary process and other toughening mechanisms inside the dactyl club under high-speed impact were uncovered.

## 1. Introduction

Lightweight, high-strength and high-toughness composite materials are continually developed and improved to meet ever-increasing performance needs in construction aerospace and defense sectors (Obradovic *et al.*, 2012 ▸; Muneer Ahmed *et al.*, 2021 ▸). However, a better balance between strength and toughness is always challenging to achieve in traditional material manufacturing, especially in producing lightweight materials (Ritchie, 2011 ▸). To address this challenge and develop materials with an excellent combination of strength and toughness under high-speed impact, scientists and engineers seek inspiration from natural materials (Huang *et al.*, 2019 ▸; Liu *et al.*, 2016 ▸; Naleway *et al.*, 2016 ▸; Wegst *et al.*, 2015 ▸; Grunenfelder *et al.*, 2014 ▸), such as scales (Zimmermann *et al.*, 2013 ▸), nacre (Yao *et al.*, 2014 ▸) and antlers (Launey, Chen *et al.*, 2010 ▸). Compared with engineering materials, the fabrication of natural materials is sustainable, and achieves a good balance between strength and toughness through excellent structural design across multiple length scales, from the molecular to the macroscopic (Huang *et al.*, 2019 ▸; Ritchie, 2011 ▸).

**Figure 1:**
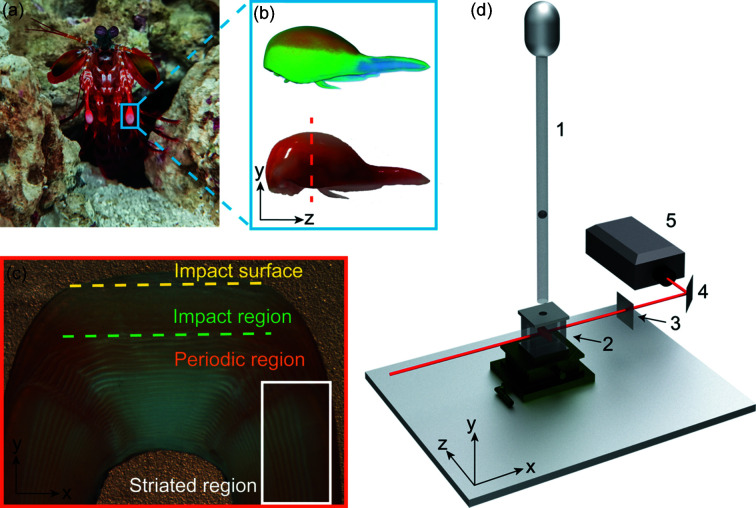
Samples and experimental setup. (*a*) Photograph of *Odontodactylus scyllarus* with dactyl club indicated (blue square). (*b*) Top: 3D rendering of dactyl club based on laboratory CT result. Bottom: photograph of the dactyl club separated from raptorial appendage. (*c*) Optical microscopy image of a cross-section of dactyl club from (*b*) sectioned along the red dashed line. The impact surface, impact region, periodic region and striated region are depicted in (*c*). (*d*) Schematic of the experimental setup for the *in situ* impact test equipped with a high-speed X-ray imaging system. 1: gas gun; 2: sample holder; 3: scintillator; 4: mirror, 5: high-speed camera.

The stomatopod dactyl club [Fig. 1 ▸(*b*)], which acts as the offensive organ during preying and ritualized fighting, is one of the toughest and most impact-resistant biological tissues in nature (Patek *et al.*, 2004 ▸; Claverie *et al.*, 2011 ▸). The exoskeletons of stomatopod dactyl exhibit remarkable tolerance against dynamic impacts which can induce catastrophic damage on the shells of prey like crab, mollusk *etc.* (Patek & Caldwell, 2005 ▸). As these creatures use the same environment to obtain the elements that make up their exoskeleton, the stomatopod must have adapted superior structure-optimization strategies to develop its dactyl clubs compared with its prey. It is therefore an ideal natural material for research into optimizing strength and toughness for high-speed impact-resistant biomimetic composites.

Extensive studies have been performed over the past ten years to uncover the structural optimization strategies and toughening mechanisms of the stomatopod dactyl, generating numerous characterization results, and various intrinsic and extrinsic toughening mechanisms have been derived (Weaver *et al.*, 2012 ▸; Amini *et al.*, 2014 ▸, 2015 ▸; Yaraghi *et al.*, 2016 ▸; Grunenfelder *et al.*, 2018 ▸; Huang *et al.*, 2020 ▸; Chua *et al.*, 2021 ▸). To date, these studies have already provided innovative inspirations for the manufacture of high-energy absorption and impact-resistant composite materials in industry (Han *et al.*, 2020 ▸; Liu *et al.*, 2019 ▸; Rivera *et al.*, 2020 ▸). One of the first comprehensive reports on stomatopod cuticles suggested they are made of three distinct regions: the impact region, the periodic region and the striated region (Weaver *et al.*, 2012 ▸) [Fig. 1 ▸(*c*)]. X-ray diffraction (XRD), energy-dispersive spectroscopy (EDS) and nanoindentation combined with dynamic finite element analysis (DFEA) were performed in this study, leading to several important conclusions: the origin of the internal cracks is located near the impact surface (the periodic region) and the oscillating elastic modulus of adjacent lamellae as well as a larger-scale modulus mismatch between different regions plays an important role in preventing and deflecting cracks. A second study (Amini *et al.*, 2014 ▸) demonstrated that the chemical and microstructural variation of the mineral distribution in the tissue periphery strengthens the dactyl. Under nanoindentation tests, the hy­droxy­apatite (HAP) crystallites tend to exhibit misalignment behaviors by sliding and rotation, showing graded, quasi-plastic mechanical responses in the outer region of the dactyl (Amini *et al.*, 2015 ▸). Recently, a further experiment-based quantitative analysis demonstrated that plastic dissipation at the crack tip is the main contribution to the fracture resistance, with both linear elastic fracture mechanics (LEFM) and elastic plastic fracture mechanics (EPFM) protocols tested (Chua *et al.*, 2021 ▸). By employing nanoindentation to initiate cracks, it was also found that the cracks can be deflected by out-of-plane fibers (Yaraghi *et al.*, 2016 ▸). Finite-element analysis and simulation tests using biomimetic 3D-printed structures suggested that the herringbone architecture in the interface of the impact surface and impact region of dactyl may offer an enhanced stress redistribution and out-of-plane stiffness in response to compressive loading on top of helicoidally structured dactyl cuticle (Yaraghi *et al.*, 2016 ▸). Another method using high-strain rates in the nanoindentation test (around 10^4^ s^−1^) demonstrated that the toughness of its hard mineral surface consisting of highly regular-arranged nanoparticles was related to the impact strain rate (Huang *et al.*, 2020 ▸). The mineral coating has exceptional energy-dissipation efficiency under high-strain rate impact, and is able to localize damage and prevent crack initiation and propagation under high-speed impact. However, the deformation induced by nano­indentation was only restricted to a small localized surface area on the dactyl.

The aforementioned structure properties and toughening mechanisms were mainly uncovered by static or quasi-static structural and mechanical characterizations. Most of the studies focus on investigating the local structure and mechanical properties of stomatopod dactyl cuticle, as opposed to *in situ* dynamic visualization, due to the limitation of the applied characterization methods. The structural–mechanical mechanisms across hierarchical levels under dynamic loading are relatively few and remain to be explored. In fact, systematic investigation and analysis on multiscale material properties of stomatopod dactyl still heavily rely on modeling, *e.g.* finite element method (FEM) and other simulation approaches. The lack of experimentally verified structure and material parameters as inputs at the extreme strain rates and loads involved undermines the credibility of the simulation results. The loading strain rate is a crucial factor that can affect the mechanical behaviors and, therefore, experimental characterization under realistic impact speed (∼25 m s^−1^) is crucial to uncover the dynamic structure–property relationship of stomatopod dactyls. In fact, we used a higher impact speed (∼160 m s^−1^) in this study to induce visible deformation within the dactyl.

To study the multiscale dynamic deformation mechanisms of dactyl club is technically challenging. Ideally a method with a high 3D spatial resolution (micrometre scale) and a large field of view is needed to examine the hierarchical-structured dactyl, and the measurement has to be fast (microsecond-level resolution). Synchrotron X-ray imaging and tomography methods are powerful and nondestructive tools enabling the probing of 2D and 3D structural properties and deformation behavior of materials (Zhang, Chen *et al.*, 2020 ▸). X-ray transmission measurements can potentially determine micrometre-level strains on a microsecond temporal-scale. On the other hand, it is highly desirable to study the mechanical behavior of the heterogeneously structured dactyl club without the ability to assess the deformation on a 3D volumetric basis. Conventional micro-computed tomography (micro-CT) can provide static 3D structure characterizations of materials from the micro- to meso-scales and, in some cases, 4D X-ray micro-CT can be used to examine the internal deformation behavior and fracture processes in different loading situations (Takeuchi & Suzuki, 2020 ▸). However, micro-CT techniques are generally not applicable for fast characterization of structures ranging from multiple length scales due to a contradiction of spatial and temporal resolutions with a desired field of view. So far, the spatial and temporal resolutions of synchrotron X-ray micro-CT techniques are far from capable to view the damage and fracture process occurring inside the whole dactyl club. Hence, a customized characterization strategy taking advantage of the merits of different methods was employed for this project.

Herein, we performed *in situ* spherical projectile penetration tests [Fig. 1 ▸(*d*)] on the stomatopod dactyl using an in-house system developed to mimic high-strain rate (∼10^6^ s^−1^) impact-loading situations encountered by the stomatopods in nature. A combination of real-time 2D X-ray imaging and high-resolution post mortem tomography examination was employed to investigate the structure evolution induced by dynamic loading of the dactyl club. Equipped with a superconducting wiggler, the 3W1 beamline of the Beijing Synchrotron Radiation Facility (BSRF) offers fast X-ray imaging capability to perform *in situ* characterization of the nucleation and propagation of cracks in 2D, while high-resolution post mortem micro-CT was used to examine the 3D internal structure in detail. We revealed the full crack evolutionary process and corresponding toughening mechanisms inside the dactyl club, and differences in mechanical responses caused by hydration variation. Furthermore, a detailed structural characterization on the impact surface and impact region using nano-CT was carried out. DFEM simulations were performed on the reconstructed structure to unveil the mechanical roles of pore canal networks, chitin fiber scaffolds and mineral particles within this critical region of the dactyl club.

## 2. Experimental

### 2.1. Materials

Live specimens of mantis shrimps (*Odontodactylus scyllarus*) were obtained from online retailers in Taobao and were frozen and stored at −80°C until use. The dactyl clubs with a smooth surface were selected for the *in situ* impact experiment. The dry dactyl used in the impact experiment were dried in a hot air oven at 30°C for 6 h. The dactyl clubs after impact were carefully refrigerated before X-ray CT examination. Finally, for the scanning electron microscopy (SEM) tests, the same samples were embedded in ep­oxy (SPI-Pon812) and then sectioned using a linear-precision diamond saw IsoMet 4000 (Buehler), ground and polished sequentially with P1200/4000/5000/7500-grade SiC paper and finally polished with 1 µm diamond paste, followed by a 10 min ultrasonic bath to remove debris from the sample surfaces (Amini *et al.*, 2015 ▸).

### 2.2. *In situ* dynamic imaging during impact

Impact experiments with *in situ* dynamic imaging on the dactyl club were carried out at the 3W1 beamline of the BSRF where an in-house setup was developed for impact penetration with *in situ* phase contrast imaging (PCI). High-flux white X-ray beams generated by a superconducting wiggler were used to illuminate the dactyl sample and a high-speed X-ray imaging system was used for fast structure characterization of the dactyl clubs. As shown in the schematic of the impact experiment setup [Fig. 1 ▸(*d*)], a dactyl sample was fixed at the sample stage waiting for the impact from an accelerated projectile with a diameter of 500 µm at a speed ranging from 110 m s^−1^ to 200 m s^−1^. During impact, the frame rate of the X-ray camera is set at 200 000 frames s^−1^, and the exposure time at 6 µs. The effective pixel size of the acquired image is about 4 µm × 4 µm.

### 2.3. X-ray computed tomography

To elucidate the crack distribution, 3D X-ray CT characterizations were performed on the same damaged samples at the X-ray Imaging and Biomedical Application Beamline (BL13W1) in the Shanghai Synchrotron Radiation Facility (SSRF). The nominal pixel size of the reconstructed image is about 3.25 µm × 3.25 µm. The X-ray energy was set at 30 keV and reconstructed into 3D images with the software *PITRE* (Chen *et al.*, 2012 ▸). Subsequently, crack segmentation and analysis were conducted using the *Avizo* software. In addition, the morphology observations of the entire structure of dactyl clubs were performed on a photon-counting detector based spectral Micro-CT developed by the Institute of High Energy Physics, Chinese Academy of Sciences.

Nano-CT characterization on the impact surface and impact region was carried out using a full-field transmission hard X-ray microscopy system installed at the 4W1A beamline in the BSRF with a beam energy of 8 keV. Finally, the volume spatial resolution is ∼64 nm. Segmentation and model meshing for DFEA were conducted with *Avizo*.

### 2.4. Dynamic finite-element analysis

DFEA of an impact event between the junction region of the impact surface and impact region with a rigid target was designed using *Avizo* and analyzed using the commercial finite-element software *ABAQUS/EXPLICIT* (Simulia, 2012 ▸). The mechanical response of the material of model I was modeled as isotropic linear elastic with different mechanical properties (*E*_1_ = 70 GPa for mineral coating and *E*_2_ = 35 GPa for fiber scaffold) and mass density (ρ_1_ = 3160 kg m^−3^ for mineral coating and ρ_2_ = 1440 kg m^−3^ for fiber scaffold). The elastic modulus of the fiber bundles in model II was set at 70 GPa. The finite element mesh contains a total of 2 137 387 elements. In these simulations, the target was set into a rigid ball with 1 × 10^−6^ m radius, 1 × 10^−16^ kg mass and 200 m s^−1^ speed. The contact between the model and target was defined as frictionless (tangential) and hard (normal).

### 2.5. Scanning electron microscopy

Whole or polished dactyl clubs were examined with a scanning electron microscope (Hitachi S-4800) at an accelerating voltage of 10 keV. To prevent surface charging, the samples were coated with Pt.

## 3. Results and discussion

### 3.1. Nucleation sites and crack deflection

**Figure 2:**
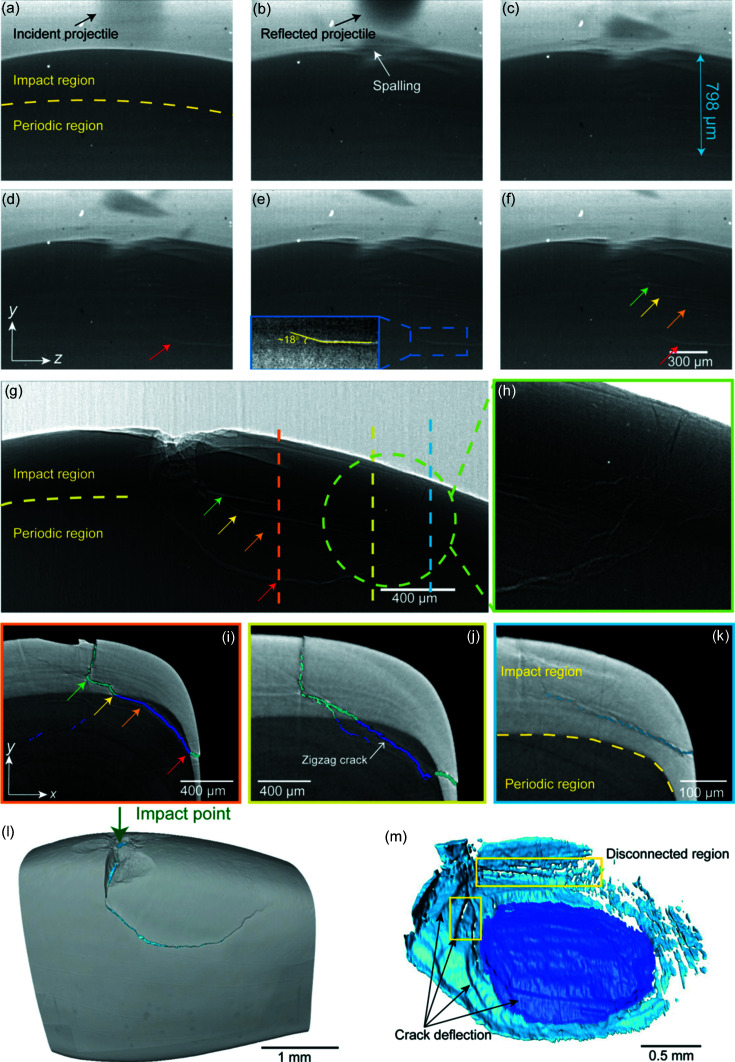
Dynamic structure deformation and crack evolution observed via *in situ* 2D fast imaging and high-resolution 3D post mortem CT examination. (*a*)–(*f*) Snapshots of dactyl club under dynamic impact with time intervals of 6 µs. Catastrophic spalling and clear crack deflections can be spotted *in situ*. Image (*e*) shows the propagating crack starting to deflect towards (highlighted with the yellow line) the impacting point at 27 µs after the exact moment of impact, which grew further [indicated by the red arrow in (*g*)] and merged with another crack system nucleated right under the impact point as shown in (*g*). Image (*g*) displays a projection of the impacted dactyl club from an *ex situ* CT test at a similar perspective as in the *in situ* 2D imaging, with some major deflection sites indicated with arrows. Image (*h*) shows crack-branching behaviors while they were deflected towards the surface of dactyl club. (*i*)–(*k*) Slices of CT reconstruction of damaged dactyl club denoted in (*g*). The main crack was deflected around the interface of the impact-periodic region. Within the periodic region, the crack was deflected periodically forming a zigzag-shaped crack path. At the end of main crack, the crack branched into microcracks and propagated to the surface of dactyl club as shown in (*k*). (*l*) 3D rendering of damaged dactyl club. (*m*) 3D distribution of the main crack system developed in the dry dactyl club sample. Dark blue: cracks within the periodic region. Light blue: cracks within the impact region.

High-speed synchrotron X-ray 2D imaging with a 5 µs super-short interval, 6 µs exposure time and 4 µm spatial resolution was employed to visualize the initiation and propagation of cracks in the stomatopod dactyl cuticle during the *in situ* impacting process [Figs. 2 ▸(*a*)–2(*f*)]. As shown in the representative images, the crack nucleation and propagation processes within the dactyl were captured while the sample was impacted by a spherical projectile with a 500 µm diameter at 160 m s^−1^. The white strips that emerged in the X-ray images featured the cracks induced by the impacting energy. As the cracks transmitted along the impacting direction, clear in-plane (indicated by the horizontal white strips) deflections toward the impact surface were initiated.

The results show that significant local fracture was mainly constrained within the impact region beneath the projectile contacting point with a length-scale of ∼160 µm. As the impacting process evolves, various damage mechanisms were observed, including sequential cleavage fracture [Fig. 2 ▸(*b*)], wing (or secondary) crack nucleation [Fig. 2 ▸(*c*)], crack deflection and coalescence [Fig. 2 ▸(*e*)] (Nomura *et al.*, 2009 ▸). Even though the contrast of the acquired 2D images is not ideal, they provide valuable *in situ* information on the occurrence and evolution of different crack systems, which is of great importance for tracing and analyzing the 3D structure information from 3D *ex situ* tomography experiments. To uncover the entire crack paths induced by the projectile impacting power, *ex situ* X-ray tomography imaging was performed on the same sample. As shown in Figs. 2 ▸(*l*) and 2(*m*), the full 3D crack systems induced by the impact were successfully captured. The projection image with a pixel size of 3.25 µm × 3.25 µm collected from the same perspective [Fig. 2 ▸(*g*)] as in the *in situ* 2D imaging offers a clearer view of the crack paths within the impact and periodic region. The cracks propagating in the different lamellae showed low coalescence [Figs. 2 ▸(*h*), 2(*k*) and 2(*m*)], along with branching [Fig. 2 ▸(*j*)] and deflecting behavior, and were guided towards the dactyl surface in the end [Figs. 2 ▸(*h*) and 2(*m*)]. Four major crack deflection sites were identified and are indicated by arrows in different colors. Figs. 2 ▸(*i*)–2(*k*) show the sliced images of the 3D reconstruction along the dashed lines in Fig. 2 ▸(*g*). The multiple nucleation sites and growth of cracks with low coalescence may lead to stress alleviation, which would be beneficial for the integrity of the dactyl club (Suksangpanya *et al.*, 2017 ▸). The four major deflection sites can be easily spotted in the CT slice image [Fig. 2 ▸(*i*), along the orange dash line]; the first major deflection (green arrow) occurred before the main crack propagated to the impact-periodic interface. This phenomenon demonstrated that the remaining impact energy is not sufficient to penetrate the fronting lamella, and is therefore inclined to spread between lamellae looking for a weak point to conquer. The crack path between the yellow and orange arrow shows that the crack traveled a long distance along the impact–periodic interface, indicating that the interface between two major regions is more favorable for crack spreading compared with the interface between two lamellae within the same region (further details are given in Fig. 1 ▸ and Note S1 of the supporting information). The results further verified that the larger modulus mismatch between impact and periodic regions (with Dundurs’ parameter of 0.33 at this interface) is important to hinder crack propagation through thickness (Weaver *et al.*, 2012 ▸; Yaraghi *et al.*, 2016 ▸). The crack path between the orange and red arrows exhibits a zigzag shape within the periodic region suggesting deflection occurred more frequently than in the impact region; this may be due to the loosely packed structure and larger pore canals in this region. The frequent crack deflection significantly delayed catastrophic fracture and increased the crack surface area, leading to improved fracture resistance (Suksangpanya *et al.*, 2018 ▸).

As shown in Figs. 2 ▸(*h*) and 2 ▸(*k*), the crack was quickly deflected back into the impact region and finally reached the dactyl surface where the remaining energy was fully dissipated. The CT slice image [Fig. 2 ▸(*j*), along the yellow dashed line] showed that crack-branching was induced in the first major deflection site. In the CT slice further away from the impacting point, the main crack turned into multiple microcrack networks, and the crack path shown in Figs. 2 ▸(*k*) and 2(*m*) appears to be discontinuous. The zigzag-shaped crack propagation path can be seen more clearly from the high-magnification SEM image [Figs. 2 ▸(*a*)–2(*d*)] collected on the cross-section of the impacted dactyl. Figs. S2(*b*) and S2(*c*) of the supporting information show how the crack penetrates through a lamella when it has enough energy, with the high stress likely tearing the fiber sheets to propagate across different lamellar layers. Separation of adjacent lamellae was also found [as shown in Fig. S2(*d*)] which provides a rare view of how the crack is propagated within the weak interface of two neighboring lamellae, where the crack path was nearly at its end, with the remaining energy consumed by the fracture of fiber bundles. It is already known (Weaver *et al.*, 2012 ▸) that, in the periodic region, the fiber bundles are arranged following the shape of dactyl clubs, while the arrangement undergoes an abrupt change in the periphery of the striated region, leading to corresponding abrupt crack propagation direction changes. The complex 3D route of crack propagation results in a significant increase in fracture toughness, and demonstrated that the overall energy absorption can be affected by the macroscale morphological characteristics (Huang *et al.*, 2019 ▸).

**Figure 3:**
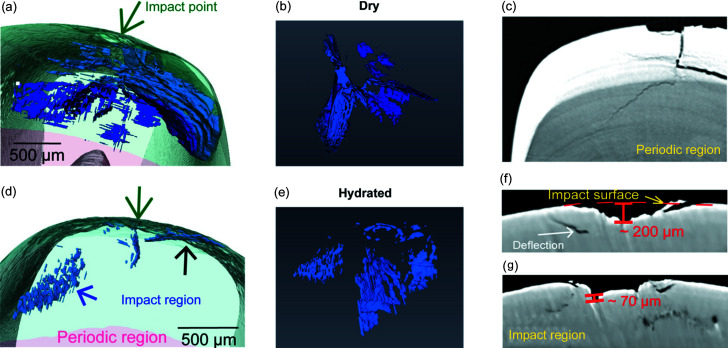
Influence of hydration state on the structural response of dactyl club subjected to impact. (*a*) 3D distribution of main crack systems within ‘dry’ dactyl club. (*b*) Crack system with a different perspective to (*a*). The isolated crack systems indicate that the crack nucleated at multiple sites. A major crack system was formed under the loading point and propagated into the periodic region, which can be easily spotted from the representative slice (*c*) of the 3D reconstruction results. A zigzag-shaped crack path can be identified in the periodic region showing a characteristic crack deflection phenomenon found in this region. (*d*)–(*e*) 3D distribution of the main crack systems within the ‘hydrated’ dactyl club. In contrast, no major cracks developed right under the loading point but several separate cracks formed around the loading point. The main cracks propagated to the interface of the impact surface and impact region and are then significantly deflected, which can also be easily spotted from representative slices (*f*)–(*g*) of 3D reconstruction.

Although equipped with a superconducting wiggler, the 3W1 beamline is based on a first-generation synchrotron source; the lack of coherence and low flux of X-rays prevent us from obtaining clearer and shorter time-interval images. For hydrated samples, the impact energy of the incident projectile is not sufficient to initiate visible cracks that can be observed by our *in situ* 2D X-ray imaging equipment. However, the hydration states of the dactyl sample are expected to play an important role in conducting its dynamic mechanical behaviors (Weaver *et al.*, 2012 ▸; Amini *et al.*, 2015 ▸). *Ex situ* X-ray tomography and SEM measurements were employed to investigate the toughening and energy-dissipation mechanisms of the dactyl club under the same loading conditions. According to the 3D crack networks, the volume of the main crack systems observed by micro-CT [Figs. 3 ▸(*b*) and 3(*e*)] generated within the sample is about 20× smaller than the dry sample under a similar impact energy. The results indicated that, for the dry samples, more energy was dissipated by brittle fracture such as crack initiation and extension, whereas for the wet sample more energy was consumed by other toughness mechanisms such as plastic deformation and fiber bridging. As shown in Figs. 3 ▸(*d*) and 3(*e*), several crack systems were nucleated at multiple sites away from the impact point, while surprisingly no main crack was found in the site directly beneath the impact point, which means there might have been a zone of plastic deformation beneath the impact point.

**Figure 4:**
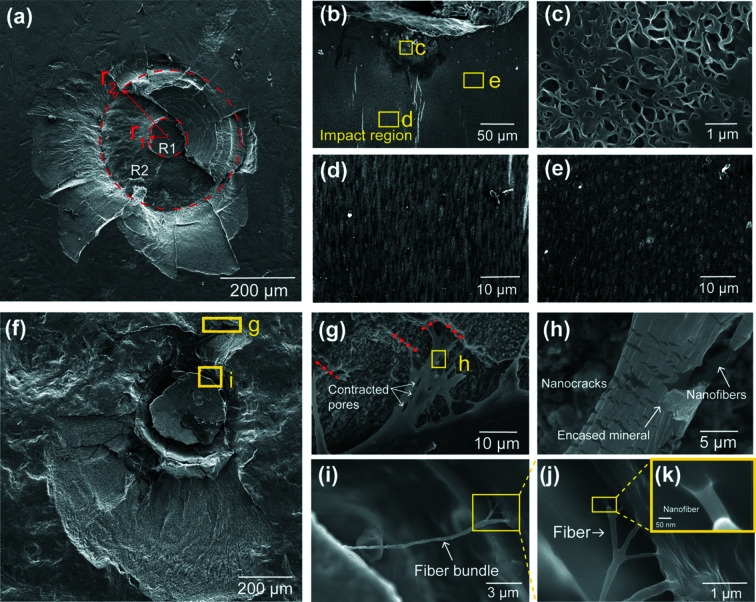
Morphologies and deformation mechanisms in the damaged dactyl surface. (*a*) SEM image of the damaged surface of hydrated dactyl club samples, impacted at a speed of 160 m s^−1^. For convenience, we labeled the surface area with the R1 and R2 regions according to a different radius from the central impacting point. (*b*)–(*e*) SEM images of the cross-section of damaged hydrated dactyl club. (*b*) Low-magnification SEM image showing both plastic and elastic regions formed beneath the impact point. (*c*) Region featuring large pores owing to the loss of mineral contents. (*d*) Enlarged view showing a typical plastic zone feature. (*e*) Enlarged view showing an elastic zone with a textured arrangement. (*f*) SEM image of a damaged surface of dactyl clubs with a rough surface. (*g*) The in-plane fibers were pulling out from different layers (highlighted with red lines). (*h*) Nanocracks in encased mineral of nanofibers and the separation of clustered nanofibrils. (*i*)–(*k*) Fiber bridging near the impact point, illustrating the hierarchical fiber bridging: (*i*) fiber bundle, (*j*) fiber and (*k*) nanofiber.

Unlike the cracks deflected around the interface of the impact–periodic region in the dry sample, the cracks within the hydrated samples deflected at the interface of the impact surface/impact region [denoted in Fig. 3 ▸(*d*) with black arrow]. Slices of reconstructed volume for the hydrated sample [Figs. 3 ▸(*f*)–3 ▸(*g*)] show the main crack deflected in the impact region and the interface of the impact surface/impact region. Distinct from the main cracks of the dry sample, the main cracks of the hydrated sample are discontinuous, as denoted in Fig. 3 ▸(*d*) with a blue arrow and in Fig. 3 ▸(*e*). The discontinuity of the crack system in the wet sample is mainly caused by the herringbone architecture in the impact region identified as a crucial structural feature for enhancing energy dissipation in the literature (Weaver *et al.*, 2012 ▸). The complex fiber orientation distribution within the herringbone structure may induce abrupt disruption against crack propagation and therefore reduce the integrity of the main crack system. The impact energy was dissipated more efficiently in the impact surface for the wet sample, hence the remaining energy was insufficient to penetrate the herringbone structure compared with the dry sample. Another toughening mechanism observed in dactyl (which is hydration-sensitive) is plasticity at the nanoscale. From SEM images of cross-section [Fig. 4 ▸(*b*)] and CT slices [Fig. 3 ▸(*f*) and 3(*g*)] of damaged hydrated dactyl club, there is a distinct plastic zone [Fig. 4 ▸(*d*)] beneath the impact point, whereas Fig. 4 ▸(*e*) shows an elastic zone with a textured arrangement. At the impact point, visualized at high magnification in Fig. 4 ▸(*c*), the existence of large pores might result from the loss of broken mineral particles. This phenomenon could be attributed to the loss of mineral particles. Under high stress, the crystallites fixed on the fibers were smashed into small irregular particles (Huang *et al.*, 2020 ▸) and exfoliated during polishing, leading to pores. Under high-speed impact, beneath the impact point the primary toughening mechanism is plasticity in a hydrated sample, which involves material phenomena like particle breakage, rotation, translation as well as microcracks, rather than crack deflection.

### 3.2. Deformation behavior of mineral nanoparticles

The impact surface [∼70 µm thick (Yaraghi *et al.*, 2016 ▸) at the outermost region of the dactyl] which mainly consists of FAP nanoparticles (Amini *et al.*, 2014 ▸) [∼60 nm in diameter (Huang *et al.*, 2020 ▸)] was considered to be mechanically crucial for providing the first shield against impact in recent studies (Huang *et al.*, 2020 ▸). Various toughening mechanisms have been found in this region from previous studies, such as reorientation of crystallites, particle breakage/rotation/translation (Huang *et al.*, 2020 ▸; Amini *et al.*, 2015 ▸). Fig. 4 ▸(*a*) shows the post-impact images of the damaged surface on the hydrated dactyl club, where catastrophic fracture and spalling occurred on the impact surface when subjected to high-speed loading (160 m s^−1^). As mentioned previously, the impact induced a plastic zone surrounding the loading point on the hydrated sample. The main fracture damage was restrained in a limited region with a diameter of 700 µm [red circle in Fig. 4 ▸(*a*) with radius labeled r_2_]. For convenience, we labeled the surface area with R1 (within r_1_) and R2 (between r_1_ and r_2_) regions according to a different radius from the central impact point. Close to the loading point where the largest impact forces were applied (R1; an area with a diameter of ∼70 µm), breakage and misalignment of mineral nanoparticles can be observed within this region [Fig. S3(*c*) within the R1 region compared with Fig. S3 ▸(*d*) within the R2 region]. The CT images [Fig. 3 ▸(*f*) and 3(*g*)] show that the deflection of the spalling occurred at a depth of ∼100 µm when it reached the interface of the impact surface and impact region. The observed phenomenon further verified the hypothesis that the unique interlocking interfaces of the two regions can enhance stress redistribution and out-of-plane stiffness, acting as an important shield to prevent the catastrophic fracture going deeper.

### 3.3. Multiscale fiber bridging behavior

Fiber bridging can effectively reduce the local stresses and strain fields at the crack tip and acts as a prevailing toughening mechanism of the hierarchical biocomposite materials (Ritchie, 2011 ▸). The association of fiber bridging with crack evolution and energy absorption inside biocomposites has been systematically studied in the literature (Launey, Buehler *et al.*, 2010 ▸; Fantner *et al.*, 2004 ▸; Bajaj & Arola, 2009 ▸; Chen *et al.*, 2008 ▸). Here, many new forms of fiber bridging behavior were captured within the impacted dactyl club.

A wider range of bridge length [tens of nanometres to micrometres, shown in Figs. S3 ▸(*b*) and S4 ▸(*f*)–S4(*k*), respectively] was examined inside the samples that suffered fast impact compared with those (on the order of micrometres) subjected to quasi-static loading (Amini *et al.*, 2015 ▸; Yaraghi *et al.*, 2016 ▸), indicating that the energy dissipation through crack bridging cannot be ignored under high-speed impact. As is known, the chitin fibrils (∼3–4 nm in diameter) self-assemble with protein to form nanofibers (∼20–100 nm in diameter). At the next hierarchical level, nanofibers are partially mineralized with calcium carbonate to form planar arrays of fibers (fiber sheets) stacked in a herringbone style structured manner to form lamellae at the microscale within the impact region (Zhang, Garrevoet *et al.*, 2020 ▸). The multiscale fiber pulling-out and bridging schemes are shown in Figs. 4 ▸(*f*)–4(*k*), offering valuable information to deduce the related energy-dissipation behavior: fiber sheets pulled from fiber lamellae [Figs. 4 ▸(*g*), S4 ▸(*b*) and S4(*c*)], fiber bundles pulled out from fiber sheets [Fig. 4 ▸(*i*)], fibers pulled out from fiber bundles [Fig. 4 ▸(*j*)] and nanofibers pulled out from fibers [Figs. 4 ▸(*h*) and 4(*k*)]. The frictional energy for pulling out fibers at different scales could all contribute to the toughening. Pulling out of fiber bundles from the unique herringbone style structured interface will be much harder as different fiber layers will be involved and easily result in large-scale twisted fiber bridging [Fig. S4 ▸(*a*)]. Also, the out-of-plane fiber bundles running through the pore canals will exert resistance. Furthermore, the pulling out process of the in-plane fibers will cause the contraction of the pore canals [Fig. 4 ▸(*g*)] and form isolated fiber sheets, along with fracture behavior of the out-of-plane fibers [Fig. S4 ▸(*d*)]. At the sites where fiber bridging is initiated, the nanofibers remain connected while the surrounding matrix is fractured and full of visible nanocracks [Fig. 4 ▸(*h*)], demonstrating a significant nanoscale toughening mechanism such as strain energy stored in the elastic fibers and the debonding energy for the fracture of mineral aggregation (Tiu *et al.*, 2021 ▸). Remarkably, high-magnification SEM images revealed how fiber bridging forms in the lower hierarchical level and its association with energy absorption. Figs. 4 ▸(*i*)–4(*k*) show remarkable fractal fiber bridging behavior near the impact point across multiple structural levels. The bridges connecting lamellae are large fiber bundles [tens to hundreds of micrometres, Fig. 4 ▸(*i*)]; at the end of the bridges, the fiber bundles split into single fibers or smaller fiber bundles [several micrometres, Fig. 4 ▸(*j*)]; further nanofibers inside those fibers are released from the encasing matrix to form the nanoscale links [Fig. 4 ▸(*k*)].

### 3.4. 3D structural characterization of the impact surface and impact region

As mentioned before, the interface and junction region between the different regions of dactyl clubs can act as crack deflectors, especially when the dactyl is under the hydration state. The complex geometry of the interfaces and enormous elastic modulus mismatch between the impact surface and the impact region might be key a structural feature for enhancing energy dissipation and crack deflection (Weaver *et al.*, 2012 ▸). However, the detailed 3D structures of this region are still relatively unknown.

**Figure 5:**
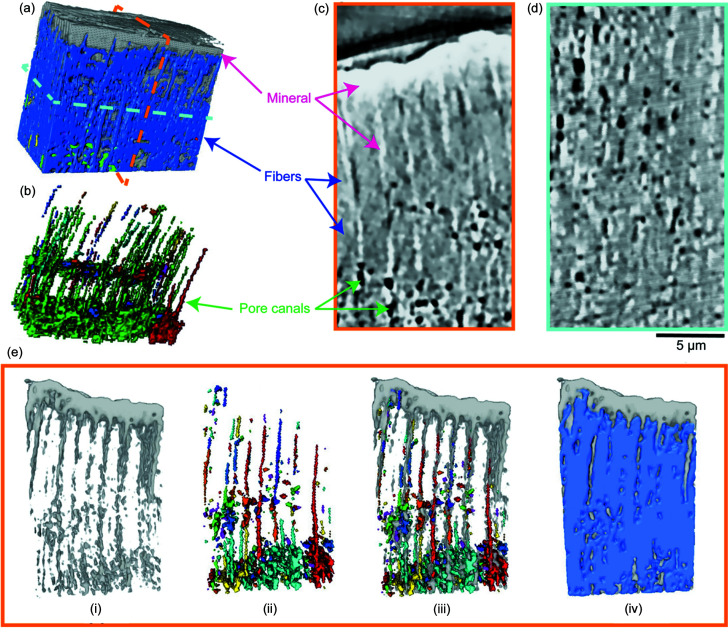
3D architecture of the impact surface and impact region revealed by nano-CT characterization. (*a*) Highly interpenetrated 3D structure consisting of three main segments: mineral nanoparticle aggregation, chitin fiber scaffold and pore canal network. (*b*) 3D model of pore canal network. (*c*)–(*d*) Slices of CT scan acquired from perpendicular projections (white regions represent mineral particles, gray regions represent chitin fiber bundles and black regions represent pore canals.). (*e*) 3D rendering of interest volume. The distributions of pore canals (*e*-ii) and minerals are highly correlated (*e*-iii) shown in colored CT slices, with (*e*-i) showing a representation of the mineral particle distribution; (*e*-iv) shows the interlocking structural design of mineral particles and fiber bundles in the impact surface and impact region.

To gain further understanding of this region, nano-CT was used to examine the structural properties in fine detail (with a 3D spatial resolution of 64 nm). The reconstructive 3D volume shows that the outermost layer of the dactyl club was coated with mineral nanoparticles and no pore canals were observed [Figs. 5 ▸(*a*)–5(*d*)]. Beneath, densely packed mineralized chitin fibers [Fig. 5 ▸(*a*)] were distributed across the impact region forming an interpenetrating pore canal network [Fig. 5 ▸(*b*)]. Surprisingly, besides the out-of-plane pore canals oriented normal to the surface of the dactyl club which were found in most regions of the cuticle by SEM (Yaraghi *et al.*, 2016 ▸; Grunenfelder *et al.*, 2018 ▸), interconnecting in-plane pore canals framework were exposed in the impact region which bundle the vertical canals together. In addition, clear evidence can be seen in the 2D slices [Figs. 5 ▸(*c*) and 5(*d*)] of the CT images that the pore canals were covered by mineral nanoparticle coating across all regions. As the 3D pore canal network is speculated to play an important mechanical role other than serving as channels for material transport during molting (Amini *et al.*, 2019 ▸, 2015 ▸), the nano-CT results furnish a new perspective of how the pore canals within the dactyl club are mechanically strengthened. The nanoparticle coatings could provide further protection to the pore canal networks from catastrophic collapse by dissipating impact energy through possible toughening mechanisms, such as particle rotation, translation and microcracks (Huang *et al.*, 2020 ▸; Amini *et al.*, 2015 ▸; Weaver *et al.*, 2012 ▸). Also, the distribution of mineral particles in different layers surrounding the pore canal networks can also act as crack deflectors resulting from the elastic modulus mismatch of the hard–soft interface (Fratzl *et al.*, 2007 ▸), thus increasing the fracture toughness of dactyl club. Due to the elastic modulus mismatch, cracks could be redirected and propagate perpendicular to the original direction, which has been summarized as a Cook–Gordan crack-stopping mechanism (Cook *et al.*, 1964 ▸). The strong correlation between the morphology of the mineral coating distribution and the pore canal networks as shown in Figs. 5 ▸(*e*-i)–(*e*-iii) also suggests that the mineralization process might be highly dependent on mineral ion supply from the pore canal systems. Moreover, a thin section of segmented 3D volume of mineral particles and fiber bundles [Fig. 5 ▸(*e*-iv)] indicates irregular suture interface design of the impact surface and impact region. These images show that, at the interface, the fiber bundles within the impact region are inserted into the mineral coating within the impact surface. Geometrically interlocking interfaces enhance interfacial stiffness and strength, and interface waviness increases resistance to crack propagation (Cao *et al.*, 2019 ▸). SEM images also illustrate the insertion of fiber bundles and mineral coating [Fig. S5 ▸(*e*)].

**Figure 6:**
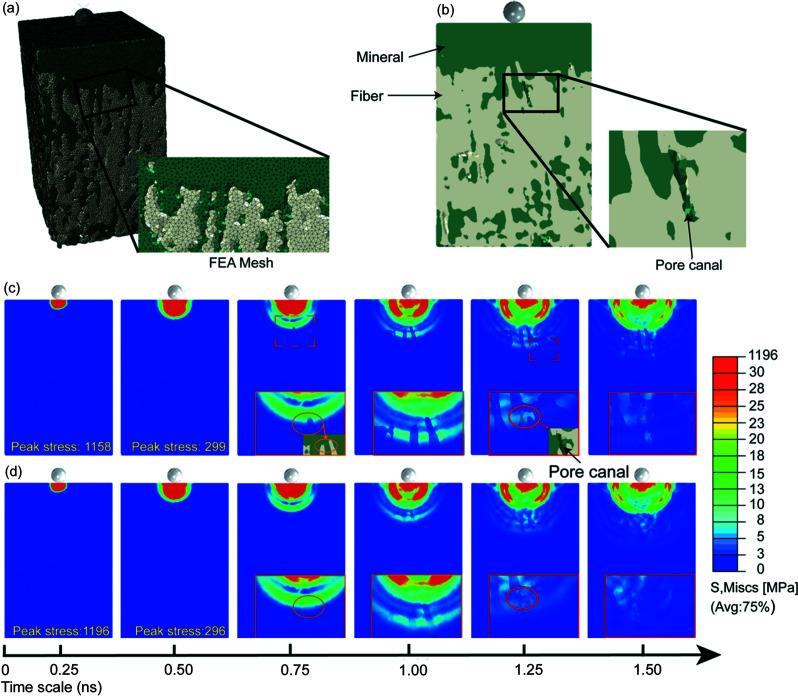
DFEM simulation for structure and mechanical analysis of the impact surface and impact region. (*a*) 3D schematic of our model following the complex microscale geometry of the impact surface and impact region. Color-coding corresponds to the different components. As shown in (*b*), the interlocking structural design of mineral particles and fiber bundles as well as the distribution mineral coated pore canals were fidelity retained in our model. Panels (*c*) and (*d*) correspond to the average von Mises stress distribution cloud diagrams of models with (*c*) mismatch and (*d*) uniform elastic modulus, respectively. The peak von Mises stress can be found in the bottom right corner of certain figures.

DFEA was performed to gain a further insight into the mechanical role of newly uncovered structures at the impact surface and impact region. The geometric shapes and dimensions used in the analytical model were based on the 3D architectural structure [Fig. 6 ▸(*a*)] acquired from the nano-CT test. Fig. 6 ▸(*b*) shows a geometric model of dactyl tissue which was converted to a 3D finite element with high fidelity. To decouple the mechanical effects between the complex nanoscale geometrical feature and elastic modulus mismatch in the interface area, two specific models were defined with different material property assignments. For model I, different elastic modulus properties were assigned to the impact surface and impact region using values reported in the literature (Weaver *et al.*, 2012 ▸); therefore, a high modulus mismatch (*E*_1_ = 70 GPa for mineral coating and *E*_2_ = 35 GPa for fiber scaffold) exists at the interface. In contrast, for model II the elastic modulus properties of mineral and fiber in the region were intentionally set to be the same in order to focus on analyzing the geometrical effects on the mechanical performance.

The impact target was set to be a rigid ball with a radius of 1 × 10^−6^ m and mass of 1 × 10^−16^ kg for both models. Figs. 6 ▸(*c*) and 6(*d*) show average von Mises stress distribution cloud diagrams of models I and II, respectively, when subjected to impacts at a velocity of 200 m s^−1^. At the initial stage (first 0.5 ns), the von Mises stress in both models was still distributed in the surrounding areas of the contact point. Peak stress (∼1150 MPa) was obtained near the impact contact zone at 0.25 ns and the value decreased significantly (∼300 MPa) in the next 0.25 ns. Notably, the initial semi-spherical shape stress pattern indicating continuous propagation was disrupted in model I immediately after the compressive wave reached the interface of the impact surface and impact region at 0.75 ns, while no clear disruption was shown in model II. The results demonstrate that the modulus mismatch in the interface acts as the first shield to hinder the impact stress propagation. As the compressive wave traveled further into the interface region, and the complex interlocking structure of the mineral coating impact surface and bulk of the impact region came into effect, the spherical shape of the stress pattern starts to exhibit obvious deformation in both models, which suggests the interlocking geometry in the interface region plays a dominant role in shielding impact waves. As the compressive wave travels deeper into the dactyl club, clear stress concentration behaviors are found around pore canal regions in model I, while no visible stress concentration signs are shown in model II (1.25–1.5 ns). The results indicated that the modulus mismatch at the pore canal region is the main reason for stress concentration. The hypothesis is further supported by the 3D mineralized pore canal networks in Fig. 5 ▸(*e*). The modulus mismatch hinders the stress and crack propagation from the mineral-coated layers into the fiber scaffolds of the pore canals, leading to the deformation and reorientation of the mineral nanoparticles and nucleation of microcracks in the mineral layer. Considering the wide distribution and large area of pore canals (specific surface area ∼7.73 × 10^5^ m^−1^) within the impact region of the dactyl club, the mineral particles in the pore canal networks would dissipate considerable energy during the impact event. The mineral particles surrounding pore canal networks help spread the damage to macroscopic tissue regions from a microscale initiation point, improving the toughness of dactyl club. This kind of mineral strengthening strategy might inspire the designs of biomedical implant materials which require high mechanical strength and microscale porosity for nutrient flow and cell proliferation (Bai *et al.*, 2013 ▸). The FEMA results demonstrated that the elastic modulus mismatches at different length scales and the irregular suture interface of the junction region can work together to prevent stress propagating into the bulk of the impact region.

## 4. Conclusions

The stomatopod dactyl cuticle is a prime example of how structural and chemical arrangement of basic biological components can create tough, impact-resistant armor to withstand high dynamic external loadings. Its exquisite hierarchical structure endows the dactyl cuticle with different energy-dissipation mechanisms. Through our *in situ* X-ray imaging measurements during a projectile penetration test which mimics the real impact situations encountered by the dactyl clubs, we experimentally observed the full process of crack initiation and evolution. Supplemented by CT and SEM results, a full 3D crack system which operates across multiple length scales was uncovered in great detail, as well as other toughness behaviors such as formation of plastic regime and fiber bridging. The real time 2D images provided critical clues in determining the time sequence of the formation and evolution of large crack systems inside the dactyl club.

The crack initiation and development mechanisms found in the *in situ* impact mechanical test experimentally verified the simulation results and hypothesis reported previously (Weaver *et al.*, 2012 ▸). The imaging and CT results show clear evidence that the crack tends to initiate at the interfaces of different regions where a high modulus mismatch exists. The cracks have a tendency for deflection at the interface when propagating across different lamellae and regions. Furthermore, the influence of hydration states on the mechanical behavior of dactyl was investigated. The dry and hydrated samples exhibit different mechanical responses under the same loading energy, and the sites of crack nucleation and the volume of the crack systems vary significantly. In addition, other toughening mechanisms including the formation of a plastic zone under the loading point, and multiscale fiber bridging systems are unique for the hydrated samples under high-speed impact around 160 m s^−1^. Moreover, a large extent of particle breakage and compaction was found beneath the contact region, which was not observed in the low strain rate nanoindentation tests (Huang *et al.*, 2020 ▸; Amini *et al.*, 2015 ▸).

A combination of nano-CT examination and DFEM study was carried out to further understand the mechanical role of the impact surface and impact region. A previously unknown heavily interlocking structure formed by multiple components in the impact surface was revealed. The mineral coating in the impact surface region and the fiber bundles within the impact region form an irregular suture like a transition zone with large tip angles (Lin *et al.*, 2014 ▸), leading to a larger area of hard–soft interface, hence is more prone to experience ductile failure and inhibit catastrophic crack propagation. Another structural feature was the unique network of interpenetrating pore canals in the impact region. A dense distribution of in-plane pore canals was found hiding in the impact region which bind the out-of-plane pore canals tightly, forming a firm interlocking system. The results also show that the 3D distributed pore canals were stiffened by mineral particles, which would more easily promote multiple initiation sites for the formation of more microcracks resulting from mismatch modulus between mineral particles and fiber scaffold.

Our results demonstrate various toughening mechanisms over a wide range of length scales that, working together, contribute to the overall dynamic mechanical properties of stomatopod dactyl clubs. Such results provide key inspirations and design guidelines for the fabrication of next-generation impact-resistant composite materials.

## 5. Data availability

All raw data that support the findings in this study are available from the corresponding authors upon request.

## 6. Related literature

The following reference is cited in the supporting information: Nixon & Aguado (2020 ▸).

## Supplementary Material

Supporting figures. DOI: 10.1107/S1600577522001217/gb5130sup1.pdf
