# Supplementary material for: In situ determination of the extreme damage resistance behavior in stomatopod dactyl club
Source: J Synchrotron Radiat. 2022 Mar 14;29(Pt 3):775–86. doi: 10.1107/S1600577522001217 (PMC9070693; doi:10.1107/S1600577522001217)
Supplement: Supplementary file 1 [file s-29-00775-sup1.pdf]

JOURNAL OF SYNCHROTRON RADIATION

Volume 29 (2022)

Supporting information for article:

In situ determination of the extreme damage resistance behavior in stomatopod dactyl club

Zheng Dong, Sen Chen, Himadri S. Gupta, Xiaoyi Zhao, Yiming Yang, Guangcai Chang, Jian Xue, Yiyang Zhang, Shengnian Luo, Yuhui Dong and Yi Zhang

Supplementary Figures

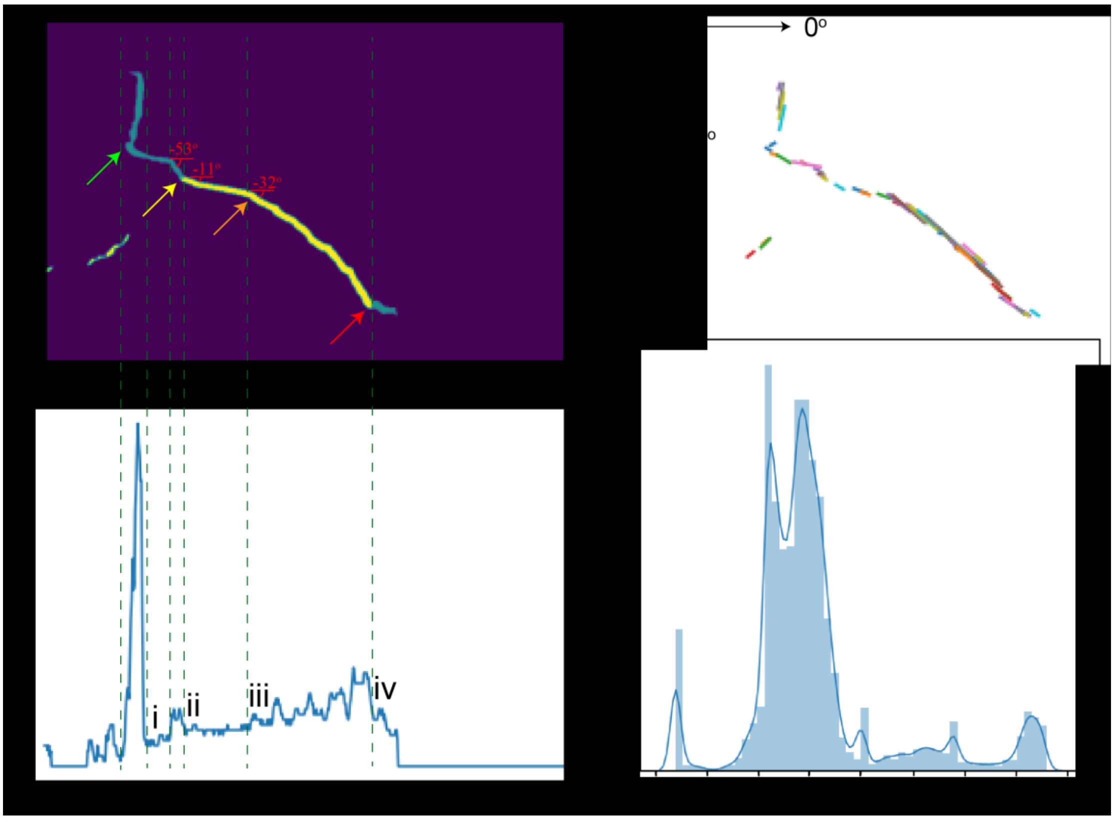

Supplementary Figure 1. Crack deflection observed via 3D post-impact CT examination. (**a**): Top: A 2D slice of the 3D volume of the crack system. (Cracks within the impact region are marked in light blue; Cracks within the periodic region are marked in yellow). Bottom: The one-dimensional integral curve of the two-dimensional cracks along the y axis. The high peaks in the curve indicate at certain x points the crack travels longer distance along the y axis. (**b**): The linearized cracks corresponding to **a**. The crack paths are extracted and linearized using the Hough transform and the local crack orientations are defined by the angles between the extracted lines and the horizontal line. The procedure was performed for all the 2D slices to get 3D statistics. (**c**): Statistical histogram of the angles of local crack orientation from the whole 3D volume.

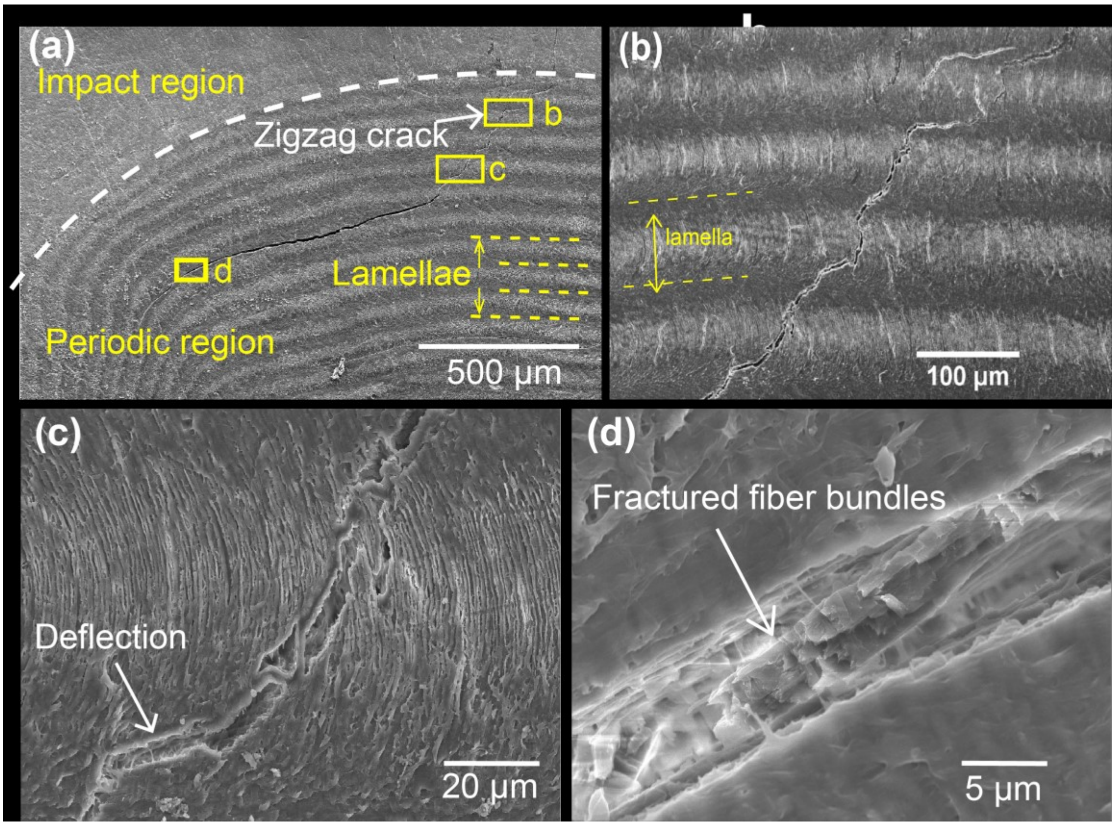

Supplementary Figure 2. Crack propagation within the periodic region. (**a**): A SEM image shows the crack propagation within the periodic region. (**b**): A zigzag-shaped path formed as the crack was frequently deflected at the lamellae interface. (**c**): Cracks penetrated through a lamella. (**d**): High magnification SEM image show fracture of fiber bundles at the crack stop site.

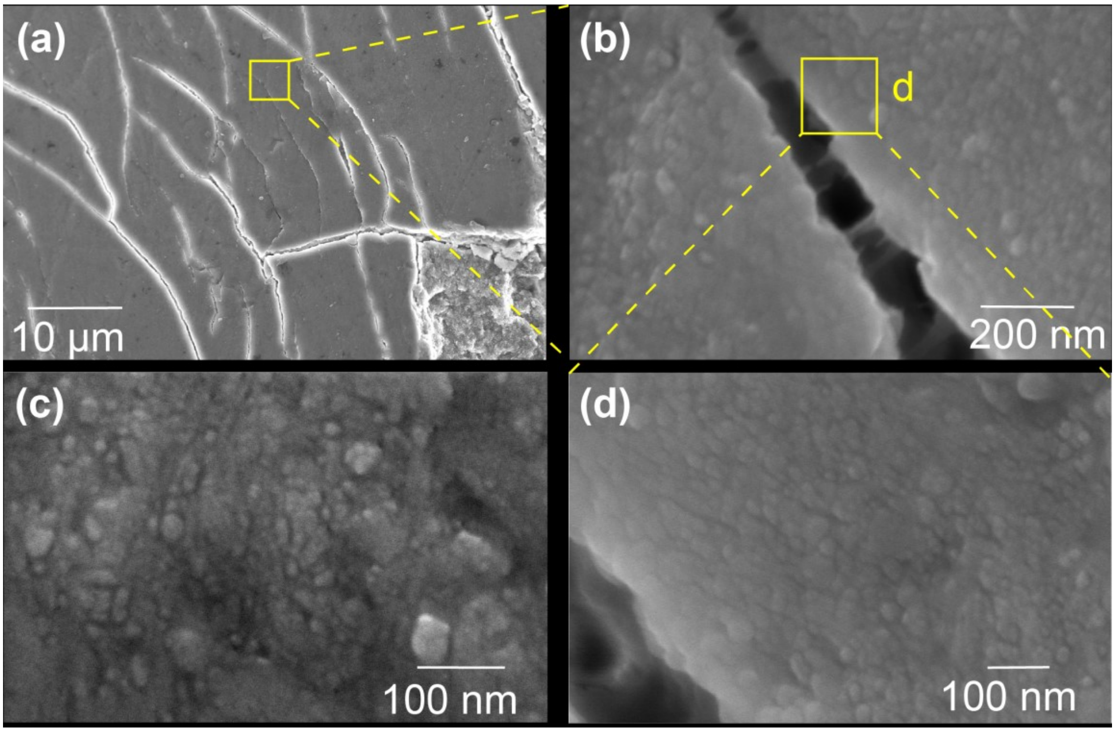

Supplementary Figure 3. Morphologies and deformation mechanisms in the damaged dactyl surface. (**a**): Plenty of circular microcracks were found on the R2 region labeled in Figure 4a. of hydrated dactyl sample. (**b**): High-magnification SEM image shows large amounts of fiber bridges existing at the crack tip. (**c**): The mineral particles were break into secondary particles at the impacted point (R1 region in Figure 4a), while they were kept relatively intact at sites away from impacted point (**d**).

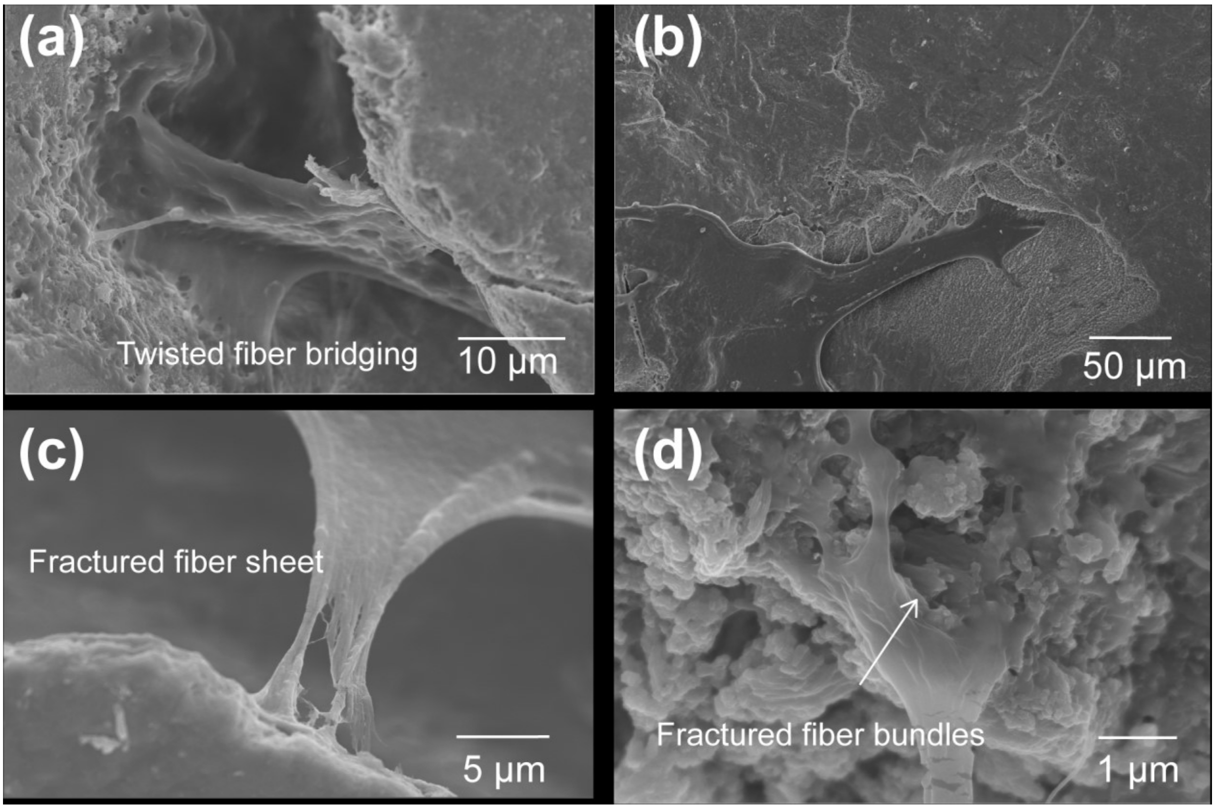

Supplementary Figure 4. Fiber bridging within the impacted dactyl club. (**a**): A twisted fiber bridge formed within a large crack which links multiple fiber layers in the helicoidal architected structure. (**b**): The fiber bridging away from impact point. (**c**): Fractured fiber sheet. **d** shows the out-of-plane fiber bundles fractured while the in-plane fiber bundles were pulling out.

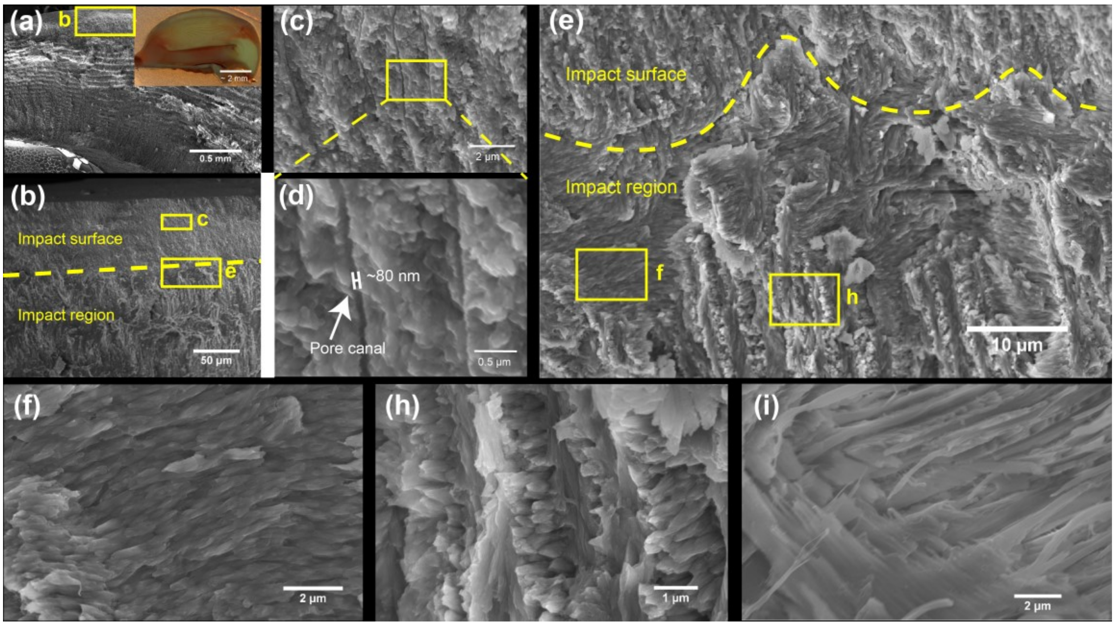

Supplementary Figure 5. Architectural design features of mineral and fibers within the impact surface of dactyl club. (**a**): SEM micrography of a cross section of a dactyl club. Inset: Optical microscopy of a cross section of a dactyl club. (**b**): The SEM micrography of the region marked in **a**. **c** and **d** are the higher-magnification SEM micrographs of impact surface of dactyl club. (**e**): SEM micrograph shows the geometrically interlocked interfaces between impact surface and impact region. (**f-h**): Fiber bundles within impact region. The fiber bundles within impact region are thicker and more densely packed compare to those in other regions. (**i**): Fiber bundles within periodic region.

Supplementary Note 1: Quantify analysis of crack propagation direction of dry dactyl club under high-speed impact.

The one-dimensional curve (supplementary Figure 1a) is the sum of the number of pixels of the crack along the y axis. Therefore, the peaks of the curve indicate the sites where the crack tends to propagate perpendicular to in-plane fiber direction. Supplementary Figure 1a illustrates that the crack prefers to travel along the impact-periodic interface between site b and site c, while the crack path between site c and site d deflects frequently. To quantitatively analysis the propagation direction of the crack system, the Hough transform is used to extract and linearize the crack paths with many lines (supplementary Figure 1b). The Hough transform is a popular extraction technique to recognize shapes in images(Nixon & Aguado, 2020). In particular, it has been used to extract lines, circles and ellipses (or conic sections), and herein, this method is used to determine crack paths in images. Furthermore, the propagation direction of a crack is indicated by the angle θ between the extraction line and the horizontal line:

$$\theta = \arctan \frac{|y_2 - y_1|}{|x_2 - x_1|} \tag{1}$$

where (x_1, y_1) and (x_2, y_2) are coordinates of the start point and the end point of an extracted line in Cartesian coordinates, respectively. The statistical results (supplementary Figure 1c) show that the angles are mainly fall into the region of −10° to −50°. As known previously(Weaver *et al.*, 2012), the in-plane fiber direction is not strict parallel to the horizontal line but followed the slight curved lamellae arrangement of dactyl clubs, demonstrating that the main cracks prefer to travel along the lamellae interface rather than perpendicular to it.

Supplementary References：

Nixon, M. S. & Aguado, A. S. (2020). *Feature Extraction and Image Processing for Computer Vision (Fourth Edition)*, edited by M. S. Nixon & A. S. Aguado, pp. 223-290: Academic Press.

Weaver, J. C., Milliron, G. W., Miserez, A., Evans-Lutterodt, K., Herrera, S., Gallana, I., Mershon, W. J., Swanson, B., Zavattieri, P., DiMasi, E. & Kisailus, D. (2012). *Science* **336**, 1275-1280.
